# A brief history of liver transplantation and transplant anesthesia

**DOI:** 10.1186/s12871-022-01904-1

**Published:** 2022-11-26

**Authors:** Sonal Sharma, Fuat H. Saner, Dmitri Bezinover

**Affiliations:** 1 Department of Anesthesiology and Perioperative Medicine, Pennsylvania State University, Milton S. Hershey Medical Center, 500 University Dr, Hershey, PA 17033 USA; 2 Department of General, Visceral, and Transplant Surgery, Medical Center University Essen, Hufeland 55, 45147 Essen, Germany; 3 Department of Anesthesiology and Critical Care, Perelman School of Medicine, University of Pennsylvania, 3400 Spruce St, Philadelphia, PA 19104 USA

**Keywords:** Liver transplantation, Transplant anesthesia, Transplantation societies

## Abstract

In this review, we describe the major milestones in the development of organ transplantation with a specific focus on hepatic transplantation. For many years, the barriers preventing successful organ transplantation in humans seemed insurmountable. Although advances in surgical technique provided the technical ability to perform organ transplantation, limited understanding of immunology prevented successful organ transplantation. The breakthrough to success was the result of several significant discoveries between 1950 and 1980 involving improved surgical techniques, the development of effective preservative solutions, and the suppression of cellular immunity to prevent graft rejection. After that, technical innovations and laboratory and clinical research developed rapidly. However, these advances alone could not have led to improved transplant outcomes without parallel advances in anesthesia and critical care. With increasing organ demand, it proved necessary to expand the donor pool, which has been achieved with the use of living donors, split grafts, extended criteria organs, and organs obtained through donation after cardiac death. Given this increased access to organs and organ resources, the number of transplantations performed every year has increased dramatically. New regulatory organizations and transplant societies provide critical oversight to ensure equitable organ distribution and a high standard of care and also perform outcome analyses. Establishing dedicated transplant anesthesia teams results in improved organ transplantation outcomes and provides a foundation for developing new standards for other subspecialties in anesthesiology, critical care, and medicine overall. Through a century of discovery, the success we enjoy at the present time is the result of the work of well-organized multidisciplinary teams following standardized protocols and thereby saving thousands of lives worldwide each year. With continuing innovation, the future is bright.

## The beginning (before 1900)

With a very long history of setbacks and successes, organ transplantation is one of the greatest medical achievements of the twentieth century. The idea of transferring body parts from one human to another is described in ancient Roman, Indian, Chinese, and Egyptian mythology. The first written mention of transplantation (skin grafting to treat burns) dates to 1550 BC [1]. The Indian surgeon Sushruta is credited with performing full thickness skin grafts in 600 BC [2]. Among the key practitioners in the history of transplantation is Scottish surgeon John Hunter (1728–1793), who performed experiments transplanting small pieces of tissue to external body sites in animals [3].

**Fig. 1:**
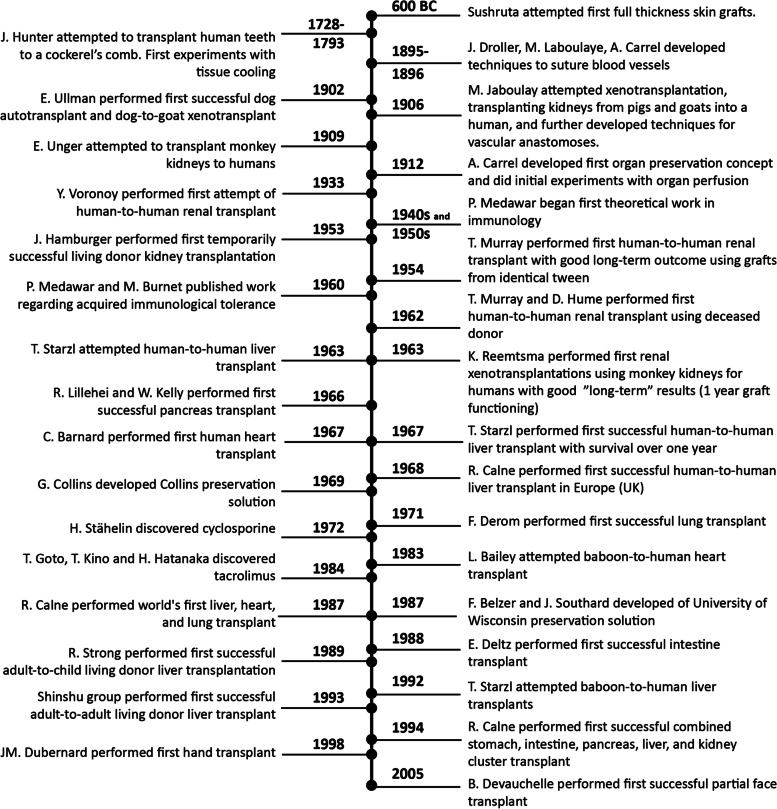
Important milestones in organ transplantation

An important step towards solid organ transplantation was taken in 1906 when Mathieu Jaboulay attempted the first xenotransplantations, transplanting kidneys from pigs and goats into humans. Unfortunately, these kidneys quickly thrombosed [4]. Later, French surgeon Dr. Alexis Carrel began work on developing preservation solutions and blood vessel suturing techniques for graft anastomoses [5, 6]. The most important milestones in organ transplantation are described in Fig. 1.

### Advances in organ transplantation (1900–1960)

Kidney transplantation (KT) was successful long before liver transplantation (LT). KT was initially performed in animals and then in humans using xenografts at first and allografts at a later stage [5, 6]. The first human renal allograft was transplanted on April 3, 1933, by Dr. Yurii Voronoy in Ukraine. He took a kidney from a 60-year-old deceased donor and transplanted it into a 26-year-old woman with mercury poisoning. Although the transplanted kidney did produce some urine, it did not provide any lasting benefit, reportedly due to ABO incompatibility, prolonged warm ischemia time (6 h), and a lack of immunosuppression therapy [5].

In the 1950s, KT became a well-established surgical procedure, but graft rejection remained common with the lack of reliable immunosuppressive therapy the principal cause. In 1954, the medical community began to see the clinical benefits of KT when a patient received a living donor kidney from his healthy identical twin brother. The surgery was performed by Dr. Thomas Murray and colleagues at Brigham Hospital in Boston. The recipient lived another eight years with his brother’s kidney. The excellent results of this transplant demonstrated the benefit of transplanting organs between twins, thereby avoiding the immunological problems responsible for causing rejection. The surgery constituted the beginning of successful clinical transplant programs.

### Beginnings of human liver transplantation

Success with KT led to increased interest in transplanting other solid organs. As a result, human LT programs began to develop in the early 1960s. Thomas Starzl attempted the first LT at the University of Colorado in 1963, which was, however, unsuccessful. The next two such surgeries were unsuccessful likewise [7]. The first “long-term” survival was achieved in 1967 when a 19-month-old girl with hepatocellular carcinoma survived for over a year following an LT [8]. In the 1980s, Dr. Starzl established the LT program at the University of Pittsburgh where surgical technique, organ preservation, immunosuppression, and medical management were refined.

Around the same time, LT programs were being established in other countries. In 1968, Roy Calne, a surgeon in Cambridge, UK, and Roger Williams, a hepatologist, started the first European LT program and reported five successful LT cases [9]. By 1970, almost 60 LTs had been performed in the United States (by Starzl) and 28 in the United Kingdom (by Calne). Within a relatively short period of time, there were 35 active transplant programs around the world.

### Transplant immunosuppression

Progress in organ transplantation would not have been possible without breakthroughs in the field of immunology in the 1940s and 1950s. An important researcher in the history of modern transplant immunology, Peter Medawar researched skin grafts during the Second World War and found that although both autografts and allografts initially healed successfully, allografts were typically rejected within two weeks. While conducting his research, Medawar encountered Burnet’s theory of immunological tolerance and went on to suggest that “self” and “non-self” are established during embryogenesis.

Over the past 60 years, many immunosuppressant agents have been introduced to clinical practice. Often, their mechanism of action was elucidated after their discovery. In addition to steroids, 6-mercaptopurine (6-MP) was one of the earliest immunosuppressive agents to be developed. It was first used in the late 1950s to suppress antibody formation in children with cancer, and Roy Calne later demonstrated that 6-MP could suppress an immune reaction in a canine kidney transplant model [10]. Gertrude Elion, a US biochemist who worked with Calne, suggested another compound that she had recently synthesized, azathioprine [11]. Not only was azathioprine superior to 6-MP in relation to preventing transplant rejection, but it was also far less toxic. Within 2 years, azathioprine was being used in human KTs along with prednisone—and both of these are still being used today [12]. Cyclosporine, an immunosuppressant isolated from the fungi *Cylindrocarpon lucidum* and *Trichoderma polysporum*, was discovered in 1972 by Dr. Hartman Stähelin. Cyclosporine depresses both cellular and humoral immunity and has a preferential and quickly reversible effect against T-lymphocytes [13–15]. Although its dose-related side effects proved to be a limiting factor in human trials performed by Calne in 1978 and 1979 [16, 17], cyclosporine received approval for clinical use in 1984. The first patient to receive cyclosporine was a 28-year-old female KT recipient. In clinical practice, cyclosporine was shown to significantly improve postoperative survival [18, 19].

Cyclosporine was routinely used for all transplantations until 1989. In the 1980s, several new medications that were more effective and boasted fewer side effects than cyclosporine and, indeed, other existing options were introduced. Discovered in 1984 by Goto, Kino, and Hatanaka, FK506 (tacrolimus: an extract of *Streptomyces tsukabaensis*) was approved for use by the Food and Drug Administration in the US in 1994. FK506 was shown to rescue 75% of the intractably rejecting human liver allografts and an equal proportion of rejecting hearts, kidneys, and other organs [20–22]. The drug, therefore, proved to be superior to cyclosporine with regard to both graft and patient survival [23]. One of the first monoclonal antibodies approved for clinical use was basiliximab. In combination with other immunosuppressive agents, it is effective in preventing rejection in both renal and hepatic grafts [24]. Extensive research focused on developing new immunosuppressive agents that will be effective in preventing rejection with minimal adverse effects is ongoing.

### Preservation solutions

Since the time of John Hunter up to the present day, the importance of effective organ preservation has been well recognized [25, 26]. In terms of the development of solutions for this purpose, their history begins with the Collins solution—a simple cold storage solution for kidneys developed in 1969 by Geoffrey Collins. This solution was modified by the Eurotransplant Foundation in 1980 to produce the Euro-Collins Solution, which was commonly used in Europe for about 15 years [27, 28].

In the late 1970s, Ross et al. developed a hyperosmolar citrate (HOC) solution (known as Marshall’s solution), which is still in clinical use today. Effective for organ preservation for up to 10 h, Marshall’s solution is used mostly for renal grafts [29, 30].

The development of the University of Wisconsin (UW) solution, introduced to clinical use in 1987, brought significant changes to transplantation practice [31]. The UW solution is quite like the Collins solution inasmuch as both use a phosphate buffer system and both have a high-potassium and low-sodium electrolyte composition. Yet, the formulations also differ from each other in important ways. In the UW solution, the glucose in the Euro-Collins solution is replaced with raffinose and lactobionic acid as metabolically inactive and osmotically active agents. Both molecules have a high molecular weight (594 and 358 Da, respectively) and counteract trans-membranous water shift to prevent cellular edema, thereby extending the preservation time for human liver grafts to more than 15 h [32].

Another popular preservative solution developed in the 1980s is the histidine-tryptophan-ketoglutarate (HTK) solution [33]. This solution was used for perfusion and flushing liver, kidney, heart, lung, and pancreas grafts as well as for hypothermic storage during transport. Its lower potassium content and the similarity of its overall composition to extracellular fluid meant this solution had a significant advantage.

### Hepatic graft shortage

Even with advances in all types of organ transplantation, LT remains one of the most critically challenging medical fields because of the difficulties encountered in treating patients with hepatic failure. Recovery without transplantation is possible in only a small percentage of cases, and liver replacement devices used as a bridge to transplant are not very effective. In recent years, improvements in surgical technique, perioperative management, and immunosuppressive therapy have yielded excellent graft and patient outcomes. Most established LT centers have a 1-year survival rate exceeding 90%, and a 3-year survival rate of over 80% [34]. Unfortunately, the need for hepatic grafts substantially exceeds their availability. Yet, this problem has been partially addressed by using split grafts, living donor liver transplantation (LDLT), and extended criteria grafts (ECG).

LDLT began in the 1980s as a strategy to deal with the limited availability of small grafts for children. The first successful adult to child LDLT was performed by Russell Strong in 1989 in Brisbane, Australia. He transplanted the left lobe of a woman’s liver into her child [35]. Just six months later, Christoph Broelsh performed the first LDLT in the US at the University of Chicago. Since then, LDLT has become a standard approach to providing liver transplants to children. The remaining donor liver and the transplanted liver in the recipient each regenerates and grows to be a fully-functioning organ as long as patient management is optimal. With a survival rate of over 90% in children, this procedure was expanded in the 1990s to include adults. In 1993, the Shinshu group performed the first successful adult to adult live donor transplant [36]. LDLT, however, is technically very challenging, requires specialized surgical training, and is associated with increased risk for both the healthy donor and the recipient. LDLT is key to transplantation success in some parts of the world such as Japan and South Korea where, until recently, deceased donor LTs were not performed due to cultural and legal barriers [37]. Currently, the number of LDLTs is growing everywhere in the world including the US, Turkey, and India—all of which have excellent postoperative outcomes [38–40].

Another way in which the pool of available organs has been increased is through the use of ECGs, which include donation after cardiac death grafts (DCD) and grafts that were initially discarded due to low quality. It has been demonstrated that both hypothermic and normothermic machine perfusion during hepatic graft preservation can improve graft quality to the extent that these organs can be used for transplantation after all [41–44]. Machine perfusion for renal grafts has been used since the 1970s [45]. And, it has been shown that hypothermic perfusion is associated with the preservation of cellular structure and mitochondrial function [41, 46] and that it produces superior outcomes in relation to the preservation of ECGs as compared with outcomes from standard cold storage [47, 48]. The primary benefit of normothermic perfusion is that physiological conditions are maintained, which has the effect of limiting endothelial damage [49]. Similar results have been demonstrated for normothermic graft perfusion [42, 44, 50, 51].

In addition, in relation to increasing the size of the donor pool, the introduction of therapy to cure hepatitis C means that hepatic grafts from hepatitis C–infected donors can now be used [52].

LT with xenotransplantation was first tried by Thomas Starzl, who made two unsuccessful attempts to transplant a liver from a baboon into a human in the early 1990s. Unfortunately, both cases were associated with severe rejection, with one patient dying after 71 and the other after 28 days. Many ethical issues must be considered and navigated in relation to xenotransplantation. Despite these challenges, research in this area has continued and is currently one of the most promising experimental programs in the transplantation context. It is likely that over time research involving regenerative medicine and advances in the science of xenotransplantation (transplantation of an animal liver into humans) will shorten the transplant wait list.

### Liver transplant anesthesia: development of a subspeciality

Dr. Jorge Antonio Aldrete served as the anesthesiologist for the first successful LT. In reference to Dr. Aldrete, Thomas Starzl in his 1992 book *The Puzzle People* wrote that “Few anesthesiologists had the skills or possessed the determination to handle these difficult cases, but it was Tony who painted the big picture. He is the pioneer transplant anesthesiologist.” In 1969, Aldrete with two co-authors who were part of the anesthesiology transplant team published a paper describing their experience in and recommendations for LT anesthesia management [53]. They point out that a complete preoperative evaluation should always be performed. However, given a lack of effective preservation solutions, these procedures were often performed as emergency procedures. As a result, it was often impossible to perform a comprehensive evaluation. The paper also provides a comprehensive description of the setting for perioperative monitoring, the medications used, and the challenges associated with LT, including in relation to correcting metabolic acidosis and managing the intraoperative administration of immunosuppression, as well as problems related to graft reperfusion and temperature management. The authors emphasize that multiple issues must be anticipated and addressed in the context of LT, including in relation to the need for heparin administration in some cases despite hepatic failure, low levels of pseudocholinesterase that begin to improve shortly after reperfusion, the effects of hypoglycemia on the development of acidosis, and the treatment of hypotension during the anhepatic phase. As the authors state, LT is not just a major abdominal surgery, but a procedure defined by a high level of complexity such that extensive specialized training is necessary in relation to the anesthesia role.

Research into transplant anesthesiology has benefited from the growing number of LT programs around the world. Much research in the field of LT anesthesia has been dedicated to the optimization of perioperative management and the standardization of anesthesia care in the stages of LT. In 1974, John Farman, in Cambridge, UK, described profound hypotension during the anhepatic and reperfusion phases of LT as well as severe hyperkalemia during reperfusion [54]. In 1987, this was described as LT-specific postreperfusion syndrome (PRS) by the Pittsburgh group [55]. and is now known to be associated with worse postoperative outcomes [56]. Hilmi et al. modified the criteria for PRS to include arrhythmias and fibrinolysis as well as persistent hemodynamic instability with the need for prolonged vasopressor support. According to a 2008 study, PRS patients required prolonged mechanical ventilation, had a longer Intensive Care Unit (ICU) and hospital stay, and were more likely than those without PRS to be re-transplanted [57].

It is recognized that nitric oxide plays a central role in cirrhosis-related vasodilation [58]. A consensus statement for the management of hemodynamic instability during liver transplantation was recently published by the International Liver Transplantation Society (ILTS), the Liver Intensive Care Group of Europe (LICAGE), and the Society for the Advancement of Transplant Anesthesia (SATA). The authors describe the primary mechanisms of hemodynamic instability in patients with end-stage liver disease and suggest treatment options for the stabilization of hemodynamics in both the systemic and portal circulation [59]. Hemodynamic management during LT continues to be one of the many challenges faced by transplant anesthesiologists.

Coagulation management of cirrhotic patients has always been a research priority for LT anesthesiologists. Using viscoelastic testing to investigate fibrinolysis during animal and human LT, Von Kaulla et al. found that hyperfibrinolysis seen in the anhepatic phase can be completely reversed with a well-functioning hepatic graft [60]. As the number of LTs increased, anesthesiologists realized that standard laboratory coagulation tests did not accurately reflect the coagulation status of patients with end-stage liver disease (ESLD). Working in Pittsburgh, Dr. Yoogoo Kang, one of the first transplant anesthesiologists, demonstrated that management of patients’ coagulation based on viscoelastic testing was associated with a decreased transfusion requirement [61]. Treatment of fibrinolysis using several antifibrinolytic agents was initially developed for LT, and viscoelastic testing has been shown to significantly help optimize the use of antifibrinolytics including in terms of avoiding thrombotic complications [62, 63]. Several other studies have demonstrated that viscoelastic testing is associated with a decrease in both red blood cell and fresh frozen plasma (FFP) transfusions. However, the need for fibrinogen concentrate, prothrombin complex, and platelets increases [64–67]. The use of viscoelastic testing in LT is also associated with significant cost savings [68]. After the first positive experiences, Thromboelastogram (TEG) became a standard coagulation monitor in the majority of LT programs. In a recent survey-based review, Schumann et al. demonstrated that viscoelastic testing is being used by 62% of all US LT programs and at 86% of large-volume centers [69]. In 2006, Klaus Görlinger published the first viscoelastic-testing-based algorithm for the management of bleeding during LT [70]. Further, the use of viscoelastic testing is recommended for coagulation management in LT [71, 72]. Viscoelastic testing for coagulation monitoring and management is now being used by practitioners in multiple subspecialities, including cardiovascular anesthesiologists, traumatologists, hepatologists, and hematologists. Several studies have shown that the use of viscoelastic testing in cardiac and trauma surgery is associated with a reduced transfusion requirement [73, 74]. It has also been demonstrated that the use of viscoelastic testing is cost-effective [75]. Although a clear survival benefit has not been demonstrated specifically with the use of viscoelastic testing, a trend towards decreased morbidity and mortality has been shown [76, 77].

In 2018, the Transplantation Committee of the American Society of Anesthesiologists published a consensus document on coagulation management during LT. Recommendations covered areas such as the administration of antifibrinolytics, the use of viscoelastic testing, the monitoring and treatment of intraoperative thromboses, and the management of patients taking oral anticoagulants [71].

LT surgery can be associated with very significant blood loss such that perioperative management of blood and blood products is another area of interest. John Sassano, a cardiac anesthesiologist, developed a rapid transfusion system capable of delivering up to 1.5L per minute. The system had a roller pump, a 3L reservoir, and an air bubble detector [78]. Commercially manufactured by Haemonetics® (Braintree, MA), it was used in LT programs until around 2000 at which time it was almost entirely replaced by an improved version made by Belmont® (Watertown, MA).

Marquez et al. demonstrated that in the absence of normal liver function, significant blood product transfusion leads to an increased serum citrate concentration (up to 20-fold) with subsequent hypocalcemia and cardiac depression. Administration of calcium chloride resulted in improved cardiac function [79].

LT frequently requires the transfusion of blood products in significant amounts, which is challenging for many blood banks. To provide the blood products required for the Pittsburgh LT program, the Central Blood Bank of Pittsburgh made major adjustments in its program encompassing improved communication, the development of new logistics, and the optimization of resources [80]. Other centers have made similar efforts [81], including promoting the use of cell salvage; putting channels for close communication in place between the transplantation program and the blood bank, which is instrumental in reserving resources ahead of time; and communicating with other blood banks in the area when a recipient has alloantibodies [80].

Hemodynamic monitoring is an important consideration in LT anesthesia. A pulmonary artery catheter was traditionally used for these cases. Transesophageal echocardiography (TEE) for LT was introduced by Dr. Andre DeWolf in Pittsburgh. In 1989, Elis et al. noted that the use of TEE helped diagnose right-ventricular dysfunction and pulmonary embolism during LT [82]. These changes were primarily observed at the time of graft reperfusion and have been implicated in the development of PRS. The concept of perioperative TEE was published in 1999 by Dr. DeWolf [83]. At present, the use of TEE for liver transplantation is increasing. Based on a US survey on the use of TEE in LT [84], the following results were reported: More than 90% of respondents stated that one or more transplant team members routinely used TEE for LT. In 38% of centers, TEE was used by all team members and in over half of the centers, by more than half the team. The respondents indicated a high level of interest in obtaining TEE certification, with 41% of TEE users reporting that they had already obtained basic or advanced certification and 12% reporting that they were in the process of obtaining certification. The survey demonstrated that over 10 years, both the use of TEE and interest in obtaining TEE certification in US transplant centers had increased significantly [85, 86]. In a position paper published in 2020, the SATA stated the opinion that TEE is both safe and effective for use during LT [87].

Since the first LT took place, the importance of a comprehensive preoperative evaluation in the selection of LT candidates has been recognized [53]. Standardization of cardio-pulmonary evaluation must be prioritized. In 1996, Plotkin et al. demonstrated that LT in patients with coronary artery disease (CAD) is associated with significant postoperative morbidity (80%) and mortality (50%) [88]. Based on this finding, the American Association for the Study of Liver Diseases (AASLD) introduced recommendations for the preoperative evaluation of LT candidates, which have since been adopted by most US and international transplantation programs. Even though a later analysis did not entirely confirm Plotkin’s results [89], the significance of CAD in LT candidates is well recognized. The optimal approach for preoperative evaluation is a topic of several current investigations.

Patients with porto-pulmonary hypertension and hepatopulmonary syndrome were initially excluded from LT. However, this changed in the 1990s after studies demonstrated the effectiveness of preoperative pulmonary vasodilator therapy as a bridge to LT, which is associated with improved outcomes [90, 91]. Much of this information became available after Susan Mandall and Michael Krowka established a national porto-pulmonary hypertension and hepatopulmonary syndrome database in 1997 [92].

Subsequently, several more LT-related database analyses became available. The United Network for Organ Sharing (UNOS) was founded in 1984 and by the early 2000s, its database contained a significant amount of data available for research. Several other national databases, as well as the Eurotransplant database, are also being studied. The use of large datasets helps answer several crucial questions. For example, in a 2009 study drawing on the Danish National Health Database, Søgaard et al. demonstrated that patients with chronic liver disease (both cirrhotic and non-cirrhotic) are prone to venous thromboembolism [93]. The results constituted a clear confirmation of a previously published (2006) study establishing an association between chronic liver disease and thromboses [94]. The mechanisms of hypercoagulability have since been demonstrated in multiple studies [95–100]. Over the years, in multiple papers based on database evaluations, researchers have endeavored to describe the perioperative outcomes of a variety of subpopulations undergoing LT, helping us to better understand several critical outcome-related questions related to perioperative thrombotic complications, mortality related to hyponatremia, and the prevalence of perioperative cardiac events.

The first protocols for Enhanced Recovery After Surgery (ERAS) specifically for LT were implemented in the 1990s. They were initially aimed at early extubation after LT. Investigators demonstrated that the use of a standardized extubation protocol was effective and safe for performing early extubation after LT [101–103]. It was also demonstrated that the “fast tracking” (early extubation and bypassing the ICU) of selected LT recipients was associated with significant cost savings and with improved one-year patient and graft survival in the fast track group [104, 105]. In a recent study, King et al. demonstrated that the use of a standardized ERAS protocol was associated with a decreased ICU stay as well as a reduction in red blood and FFP transfusion [106]. Most recently, the ILTS initiated a project to systematically evaluate a variety of multispecialty ERAS protocols for LT. Their findings will be published in *Clinical Transplantation*.

The literature includes studies, mostly animal studies although some on humans, in which researchers demonstrate that anesthesia can have a protective effect on graft outcomes. It has been shown that the use of volatile anesthetics with low hepatic metabolism, such as sevoflurane or desflurane, is associated with decreased ischemic reperfusion injury (IRI) and improved early-graft survival [107–109]. In another study, the use of sevoflurane and desflurane was associated with lower lipase and C-reactive protein levels, which indicates decreased inflammation in the allograft as compared to when isoflurane is used [107]. On a molecular level, the protective effect of volatile anesthetics on allograft survival could be related to the prevention of mitochondrial permeability transition pores, which is an important component in the IRI cascade [109]. In addition, the prevention of glycocalyx degradation, which is important for endothelium protection, has been demonstrated for some volatile anesthetics [109]. Further, the results of some studies suggest that nitric oxide donor agents may have a role in IRI treatment in recipients of hepatic and pulmonary grafts [110]. These results need further evaluation. Further, it is likely that the use of intravenous anesthetics, specifically propofol, also has a protective effect against oxidative stress and IRI [111]. Another preconditioning effect of propofol, i.e., apoptosis attenuation mediated by K-adenosine triphosphate, can potentially decrease IRI [112]. It has been suggested, too, that propofol may decrease the prevalence of acute kidney injury after LT due to the inhibition of the connexin 32 (Cx32) function and the upregulation of the erythroid-2 related factor 2 [113–115]. The results of these investigations, however, have yet to be confirmed in human trials.

Another modifiable factor that can improve graft survival is perioperative glucose control. Glucose metabolism and specifically postreperfusion hyperglycemia is a frequent problem during LT. DeWolf et al. demonstrated in an animal study that it is partially related to gluconeogenesis in the graft after liver rewarming [116]. Mallet et al. demonstrated that persistent hyperglycemia is related to decreased reuptake in the new graft and that this is a possible sign of organ dysfunction [117]. It has been shown that tight glucose control in diabetic patients can help to prevent delayed graft function after KT [118].

### Anesthesia transplantation societies

The International Liver Transplantation Society (ILTS) is a unique and rapidly growing organization that unified several specialties involved in LT. Early on, it became clear that this field of medicine can be effective only if there is a dedicated multidisciplinary team managing these very complex cases. A group of transplant anesthesiologists and critical care physicians under the leadership of Dr. Yoogoo Kang organized a multidisciplinary forum in order to discuss problems related to LT. The idea was to improve communication between clinical services and scientists as well as to optimize the coordination of LT-related projects. Held in Pittsburgh in 1984, the first meeting, “Perioperative Care in Liver Transplantation,” attracted 150 participants [119]. In 1998, the decision was made to formally name the organization the International Society for Perioperative Care in Liver Transplantation. In 1990, however, the organization changed its name to become, as it is known today, the International Liver Transplantation Society (ILTS). Since 2000, the ILTS has held an annual meeting alternating sites between North/South America, Europe, and Asia. Initially, the ILTS published congress reports in *Transplantation Proceedings*. However, given the overwhelming success of ILTS meetings and an increase in the number of LT centers, the decision was taken to start a new LT-related journal. The first issue of *Liver Transplantation and Surgery* was published in January 1995. Its name was changed to *Liver Transplantation* in 2000 [119]. Currently, the ILTS is affiliated with *Transplantation* journal*.*

John Farman, an anesthesiologist and intensive care physician, was a member of the Roy Calne program in Cambridge, UK. He had the idea of establishing a society in Europe that would be similar to the ILTS in terms of its mission. In 1987, Farman and Yoogoo Kang agreed to establish a new organization: the Liver Intensive Care Group of Europe (LICAGE). Unfortunately, John Farman passed away shortly after the organization was established. Yet, LICAGE has flourished under the leadership of anesthesiologists Michael Lindop, Gilbert Park, and John Klinck.

In 2016, the Society for the Advancement of Transplant Anesthesia (SATA) was established in the US as a national organization for anesthesiologists and intensivists involved in transplantation medicine. Currently, SATA is affiliated with *Clinical Transplantation.*

All three societies, ILTS, LICAGE, and SATA, work closely together, holding joint meetings and pursuing shared projects.

### Dedicated transplant anesthesia teams

The first dedicated team for LT anesthesiology was created in Pittsburgh in the 1980s. It became apparent at this time that the complexity of LT requires a special level and specific kind of expertise [120]. Over time, the majority of anesthesiology departments at US transplant centers have established their own transplant divisions. The introduction of specialized transplant anesthesia teams allows for the optimization of care to improve patient outcomes. Hevesi et al. demonstrated that after a dedicated anesthesia team was established, transfusion rates for blood products during LT, the postoperative need for intensive care, and the duration of postoperative ventilation all decreased significantly [121]. In a recent study, Hofer et al. demonstrated that the level of experience of the anesthesiologist caring for a patient undergoing LT has a direct effect on the patient’s outcome [122].

Subspecialty training in LT anesthesia is currently available in the US. In fact, fifteen programs offer a structured LT fellowship. In other countries, some LT programs have local fellowship opportunities. India, for example, has begun a national fellowship program similar to that available in the US. SATA recently published a position paper describing its criteria for the optimal LT fellowship structure and evaluation. The SATA Anesthesia Fellowship Task Force developed a standardized fellowship structure that includes an approach to training for core competencies based on proficiency milestones [123].

## Conclusion

The current state of organ transplantation is the result of a century of significant developments in the fields of surgery, anesthesia, medicine, and critical care. From the relatively simple concept of the anastomosis of blood vessels to complex questions pertaining to immunology and immunosuppression, the successes we enjoy today are a result of the dedication of many individuals and teams. Liver transplant anesthesia as a subspecialty is now firmly established as an integral part of any LT program. It is also the case that the expertise gained managing these patients in the operating room has enhanced patient care in many other areas of medicine.

## Data Availability

All materials used for the preparation of this manuscript are publicly available. The datasets generated during the current review are available from the corresponding author.

## Abbreviations

CAD: Coronary artery disease
DCD: Donation after cardiac death graft
ECG: Extended criteria graft
ERAS: Enhanced recovery after surgery
ESLD: End-stage liver disease
FFP: Fresh frozen plasma
HOC: Hyperosmolar citrate
HTK: Histidine-tryptophan-ketoglutarate solution
ICU: Intensive Care Unit
ILTS: International Liver Transplantation Society
KT: Kidney transplantation
LDLT: Living donor liver transplantation
LICAGE: Liver Intensive Care Group of Europe
LT: Liver transplantation
PRS: Postreperfusion syndrome
SATA: Society for the Advancement of Transplant Anesthesia
TEE: Transesophageal echocardiography
UNOS: United Network for Organ Sharing
UW solution: University of Wisconsin solution
